# Early economic evaluation of tissue engineered vascular access grafts for hemodialysis

**DOI:** 10.1080/17460751.2026.2726833

**Published:** 2026-09-21

**Authors:** Hanna de Groot, Paul J. Besseling, Chris van Lieshout, Martin Teraa, Patricia Y. W. Dankers, Marianne C. Verhaar, Renske M. T. ten Ham

**Affiliations:** a Julius Center for Health Sciences and Primary Care, Department of Epidemiology & Health Economics, University Medical Center Utrecht, Utrecht University, Utrecht, The Netherlands; b Department of Nephrology and Hypertension, Regenerative Medicine Center, University Medical Center Utrecht, Utrecht, The Netherlands; c Department of Vascular Surgery, University Medical Center Utrecht, Utrecht, The Netherlands; d Laboratory of Chemical Biology, Department of Biomedical Engineering, Eindhoven University of Technology, Eindhoven, The Netherlands; e Institute for Complex Molecular Systems, Eindhoven University of Technology, Eindhoven, The Netherlands

**Keywords:** Early economic evaluation, vascular access, tissue engineered vascular access grafts, hemodialysis, translation

## Abstract

**Aims:**

Vascular access (VA) is essential for hemodialysis in patients with end-stage kidney disease. Expanded polytetrafluoroethylene (ePTFE) grafts are used for VA but are associated with high intervention rates. Tissue-engineered vascular grafts (TEVGs) are emerging as a promising alternative. However, performance and cost thresholds for TEVGs to become clinically and economically competitive to ePTFE grafts remain unclear. This early economic evaluation aims to quantify the clinical and economic impact TEVGs must achieve to be viable in routine hemodialysis care.

**Methods:**

The Dutch VA care pathway was mapped to construct a Markov model simulating intervention rates and costs of patients receiving an ePTFE graft or TEVG. As mature clinical-effectiveness data are not yet available, model inputs were derived from literature and expert opinion. Scenario analyses explored best-case and conservative TEVG performance estimates. Sensitivity analyses identified key cost-drivers.

**Result:**

Under interview-based performance estimates, TEVGs annually yielded 0.44 fewer interventions and cost savings of €601 per patient. Thrombectomies, PTA procedures and TEVG unit price were cost drivers.

**Conclusions:**

TEVGs could become clinically and economically competitive if they substantially reduce PTA and thrombectomy rates and are manufactured at cost-efficient unit prices. These findings provide actionable thresholds to guide TEVG development and support translation.

## 1. Introduction

Kidney disease is projected to become the fifth leading cause of life years lost in 2040 worldwide [1]. Over 2.5 million people globally suffer from end stage kidney disease (ESKD) leading to hemodialysis [2]. Hemodialysis requires vascular access (VA), but current VA options have high complication rates, including stenosis, thrombosis, and infections. These complications lead to VA dysfunction, recurrent surgical and endovascular interventions and increased morbidity, mortality, and healthcare costs.

Current VA options include autologous arteriovenous fistulas (AVF) and synthetic arteriovenous (AV) grafts [3]. AVFs, created by connecting a native artery to a vein, are preferred for their optimal patency rates and low complication risk [4,5]. However, many patients are ineligible for AVF creation due to unsuitability of native vasculature or high risk of non-maturation, both linked to advanced age or preexisting disease [3]. Non-maturation occurs in nearly one-third of AVF cases and often requires re-interventions [6,7]. Furthermore, AVF maturation requires 4–6 weeks, during which temporary central venous catheters (CVCs) are required. CVCs carry additional risks, including (bloodstream) infections and central venous stenosis [8]. An alternative to AVFs is synthetic AV grafts, typically made from expanded polytetrafluoroethylene (ePTFE). These ePTFE grafts can be used to create VA without relying on native vasculature and do not require a maturation phase. However, re-intervention rates are higher due to an increase in intimal hyperplasia, resulting in lower patency rates than AVFs [9].

To overcome limitations of current VA options, advances in the field of regenerative technologies have led to tissue engineered vascular access grafts (TEVGs) [10]. TEVGs aim to mimic advantageous properties of native vasculature, potentially reducing (re-)interventions compared to ePTFE grafts, while being readily available for patients ineligible for AVF creation [11]. TEVGs are tubular structures composed of biodegradable and/or (human) extracellular matrix materials. They can be categorized into three types based on their production method [12–17]: 1) *in vitro* TEVGs are fabricated in a controlled laboratory setting prior to implantation; 2) *in vivo* TEVGs are formed subcutaneously within the patient’s body, and later harvested and implanted as a VA graft; and 3) *in situ* TEVGs are implanted directly, whereafter the grafts are gradually repopulated by the patient’s own cells [13,15,18–20]. TEVGs are progressing toward advanced clinical developmental stages with recent (pre-)clinical results showing great promise [13,15,18–20]. However, for clinical adoption, TEVGs must also be clinically and economically competitive with current VA options.

Translating (bio)medical technologies faces scientific, technical, organizational, and economic challenges [21,22]. For TEVGs, these include uncertainty about physiological effects, production timelines, and costs [23]. Early economic evaluations aim to guide the development and implementation of emerging technologies, especially when uncertainties remain. In the absence of mature clinical data, scenario analyses can be used to define thresholds for minimum clinical performance and maximum costs, by quantifying potential clinical benefits and costs-consequences. These thresholds generate insights into feasibility, value and translational potential of technologies, enabling developers to anticipate obstacles and mitigate risks during clinical translation [24,25].

This study defines clinical and economic thresholds TEVGs must meet to compete with ePTFE grafts in VA care of ESKD patients undergoing hemodialysis in the Netherlands. ePTFE grafts were selected as the comparator based on expert opinion, as they are likely the first to be replaced by TEVGs in clinical practice. Findings aim to guide developers and researchers in further TEVG development, while also informing payers, clinicians, and other stakeholders about the potential value of future clinical adoption.

## 2. Methods

### 2.1. Study design and population

An early economic evaluation compared intervention rates and costs of ePTFE grafts and TEVGs. Anticipated clinical effects and costs for three TEVG types (*in vivo, in vitro* and *in situ)* were informed by expert opinion and grouped in the primary analysis. Rather than providing firm evidence on TEVG cost-effectiveness, the analysis aims to estimate the clinical and economic thresholds for TEVGs to compete with ePTFE grafts. To do so, potential variation in clinical effects and unit costs of TEVGs was explored through best-case and progressively less optimistic clinical scenarios at varying TEVG unit prices. The target population was ESKD patients requiring hemodialysis who are unsuitable for AVF creation. ePTFE grafts were chosen as the comparator because their application closely resembles TEVGs, making them the most likely VA option to be replaced by TEVGs.

To inform model structure, a patient journey identified care utilization across medical specialties involved in Dutch VA care. A state-transition Markov model simulated graft implantation and management. Using this model, intervention rate (including infection treatment, percutaneous transluminal angioplasty (PTA), radiological thrombectomy, stenting and surgical revision) and total costs were calculated over 12 months in monthly cycles. The time horizon of 12 months was selected as this is a frequently reported clinical endpoint in the vascular access literature, enabling benchmarking against published patency rates. Informed by expert opinion, intervention rate was used as the clinical outcome measure to reflect graft performance. The evaluation took a Dutch health care perspective and assumed budget-neutral introduction of TEVGs. This assumption reflects that VA technologies are reimbursed as part of a broader diagnosis-related group and therefore supports its adoption within existing budgets.

### 2.2. Patient journey

A patient journey provides insights into the sequence of care events in a specific care context [26]. Constructing a patient journey is particularly helpful when care includes multiple medical specialties. In this work, the patient journey was informed by Dutch and European clinical guidelines, as well as the American Kidney Disease Outcomes Quality Initiative [3,27,28]. The Dutch care pathway largely aligns with those in other western countries and can therefore serve as a suitable example for VA care in broader contexts. The patient journey visualizes what care is provided by whom (e.g., medical specialty) and where (e.g., care setting). The patient journey included care consumption during graft surgery, monitoring and management.

#### 2.2.1. Validation

To supplement and validate the patient journey, 45–60 min in-person or online semi-structured interviews were conducted with nephrologists, vascular surgeons, radiologists, dialysis nurses and patients. Stakeholders were recruited via the network of clinical partners and snowball sampling. Interviews were organized until all stakeholder groups were included and information saturation was reached [29]. Suggested changes were incorporated.

### 2.3. Early cost-effectiveness model

#### 2.3.1. Model design

A Markov model was constructed to simulate care consumption and associated costs of VA creation and maintenance with either an ePTFE graft or TEVG. The patient journey informed model design, which consists of five states: graft surgery, functional graft, intervention, graft abandonment and death (Figure 1). The model structure was adapted from Alarid-Escudero et al. and built in RStudio (2023.03.1, Build 446) using the dampack package [30]. The script is available from the authors upon reasonable request.

**Figure 1.**
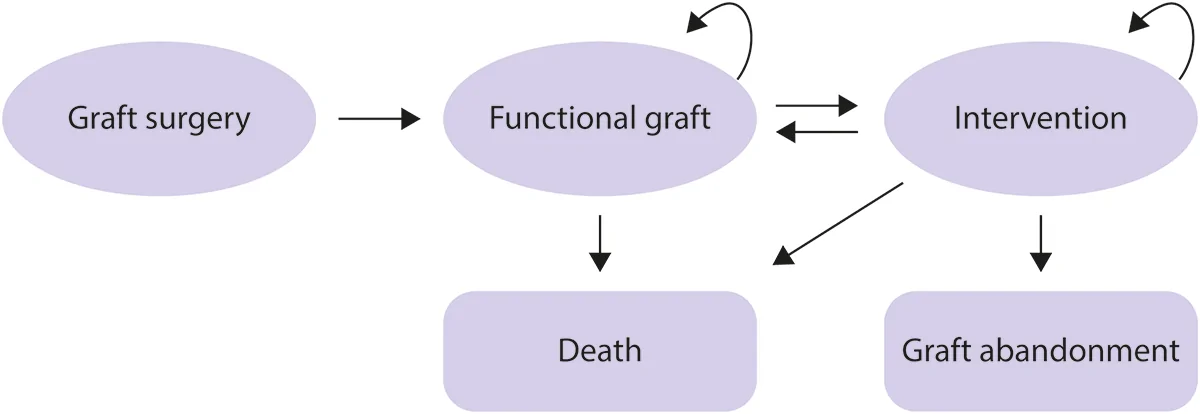
Schematic representation of the model structure.

All patients enter the model via graft surgery of ePTFE grafts or TEVGs, whereafter all patients move to the functional graft state. From there, patients can cycle to the different states according to the arrows. Rectangle-shaped states are absorbing states. Interventions include infectious treatment, percutaneous transluminal angioplasty, radiological thrombectomy, stenting and surgical revision.

Abbreviations: ePTFE = expanded polytetrafluoroethylene, TEVG = tissue engineered vascular access grafts.

At model entry, a hypothetical cohort of patients received an ePTFE graft or TEVG via graft surgery and progressed to the functional graft state. Each month, patients could remain in the functional state (indicated by the circular arrows) or transition to the intervention or death state. A successful intervention in the intervention state returned patients to the functional state, while failure led to re-intervention or graft abandonment. Grafts were not surgically removed after graft abandonment. The death or graft abandonment health states mark the end of the graft’s lifespan (‘absorbing states’).

#### 2.3.2. Intervention rates and effectiveness measures

Monthly transition probabilities determined how the cohort progressed through the different states. Transition probabilities for ePTFE grafts were informed by literature, while TEVGs’ transition probabilities were informed by a combination of literature and expert opinion as sufficient clinical data is lacking [9,31,32]. Experts were consulted via 17 semi-structured interviews with clinical experts to elicit anticipated changes, both positive and negative, in intervention frequency when using TEVGs compared to ePTFE grafts. As experts encountered difficulties providing estimates for individual TEVG types, the three TEVG types (*in vivo*, *in vitro*, and *in situ*) were grouped. Interviews followed a structured elicitation procedure. Experts were presented with each complication separately and asked whether they anticipated any effect on complication rates relative to ePTFE grafts. If an effect was anticipated, they were asked to indicate the direction (more or fewer interventions) and magnitude of the expected change. Base case parameters were selected based on the most frequently reported expectation per complication type. Based on these inputs, a combined base case scenario for all three types of TEVGs was formulated in which the frequency of treatment of infection and PTA procedures were reduced by 50% compared to ePTFE grafts. This selection was triangulated with experts outside the original interview panel. Transition probabilities for the base case were calculated (Table 1). Model outcomes were validated by two clinical experts.

**Table 1.** Transition probabilities used in the Markov model for expanded polytetrafluoroethylene (ePTFE) or tissue engineered vascular access grafts (TEVG).

Transition probabilities			
	ePTFE	TEVG	Source
Functional → Intervention	0.267	0.195	[9], Expert Opinion
Functional → Death	0.011	0.011	[32]
Intervention → Functional	0.533	0.533	[31]
Intervention → Death	0.067	0.067	[31]
Intervention → Graft abandonment	0.12	0.12	[31]

#### 2.3.3. Costs

The patient journey informed monthly care consumption. Care consumption included graft surgery, hospitalization, antibiotics and surgical or endovascular interventions (Table 2). Costs were derived from the Dutch costing manual, price lists from the Dutch National Healthcare Authority (Nederlandse Zorgautoriteit; NZa) and literature [31,33,34]. Unit costs for ePTFE grafts were based on internal hospital prices. TEVGs’ costs were estimated based on expert opinion. Prices were converted to 2023 euros (€) [35]. No costs were assigned to graft abandonment or death, as these mark the end of the graft’s functional lifespan and fall outside the scope of VA creation and maintenance.

**Table 2.** Costs used in the Markov model for expanded polytetrafluoroethylene (ePTFE) grafts or tissue engineered vascular access grafts (TEVG).

Costs		
	Costs	Source
***Device unit costs***		
Graft material (ePTFE)	€ 976	Expert Opinion
Graft material (TEVG)	€ 1 000	Expert Opinion
***Intervention costs***		
Graft surgery	€ 1 319	[33]
Hospitalization	€ 547	[34]
Surgical revision	€ 1 080	[31]
Percutaneous transluminal angioplasty	€ 1 992	[33]
Thrombectomy	€ 3 180	[33]
Stenting	€ 3 779	[33]
Antibiotics	€ 7	[31]
Blood tests	€ 20	[31]
Duplex	€ 125	[33]
***Routine healthcare costs***		
Flow measurement	€ 38	[34]

Costs are grouped by device unit costs, intervention costs and routine management costs.

#### 2.3.4. Assumptions

Several assumptions were necessary due to limited availability of data and model simplicity, consistent with early health economic practices. Two clinicians validated assumptions, model structure and input parameters. First, one major assumption is the unit cost for TEVGs. This assumption represents an order-of-magnitude approximation of procurement costs rather than a precise estimate, given the absence of established market pricing. ePTFE graft and TEVG surgeries were assumed similar, with equal costs. Furthermore, all graft surgeries were assumed to be successful. The simulated cohort was modelled to reflect the mortality rates of the general Dutch dialysis population [32]. Additionally, monthly probabilities of interventions were assumed to remain constant over time. Patients who contracted an infection were modeled as 3-day hospitalization plus 6 weeks of oral antibiotics [31]. Routine flow measurements were assumed to be performed monthly and costed as a 15-min nurse consultation [36].

### 2.4. Scenarios and sensitivity analyses

Given the limited availability and uncertainty of data on intervention rates and costs of TEVGs, four scenarios were explored in addition to the base case (Table 3). Scenario 1 represents a best-case scenario in which TEVGs achieve similar intervention rates as AVFs, the current gold standard [9]. To reflect this, intervention rates reported for grafts were reduced by 63% for infectious treatment (from 0.10 to 0.04), 28% for PTA procedures (from 1.84 to 1.33), 96% for thrombectomies (from 1.13 to 0.04) and 45% for surgical revisions (from 0.31 to 0.17), based on reported ePTFE graft and AVF data [9]. Scenario 2–4 simulated progressively less optimistic outcomes, applying 50%, 25% and 10% of the reductions defined in scenario 1. Stenting was not included in scenario analyses, as it occurs more frequently in AVFs. Calculations for scenario-specific complication rates are provided in Supplementary Table S1.

**Table 3.** Overview of scenarios.

Scenario descriptions									
	Infection treatment		PTA		Thrombectomy		Surgical revision		
Scenario	Reduction	Rate	Reduction	Rate	Reduction	Rate	Reduction	Rate	Source
1 (best-case)	63%	0.04	28%	1.33	96%	0.04	45%	0.17	[9]
2 (50% of best-case)	31%	0.07	14%	1.58	48%	0.59	23%	0.24	[9]
3 (25% of best-case)	16%	0.08	7%	1.71	24%	0.86	11%	0.28	[9]
4 (10% of best-case)	6%	0.09	3%	1.79	10%	1.02	5%	0.30	[9]

The best-case scenario reflects the required reduction of graft intervention rates to equal those of autologous arterio-venous fistulas (AVF). Scenario 2–4 represent progressively less optimistic scenarios, applying 50%, 25% and 10% of the reductions compared to the best-case scenario.

Abbreviations: PTA = percutaneous transluminal angioplasty.

To identify the range of TEVG unit prices at which the VA care pathway remains budget-neutral, the maximum acceptable price was explored for each scenario. TEVG unit prices were varied from €1 000 upward until cost savings from reduced intervention rates no longer offset the graft unit price. Intervention frequency and total costs were calculated over 12 months.

Uncertainty around individual base case parameter estimates was assessed via a deterministic sensitivity analysis (DSA). The DSA explores the impact of individual parameters on model outcomes by varying input values one by one between pre-set minimum and maximum values. The DSA focused on parameters differing between ePTFE grafts and TEVGs, specifically graft unit costs and intervention rates. Each parameter was varied between 50% and 150% of its base case value, and the effect on intervention rates and costs was calculated.

### 2.5. Probabilistic sensitivity analysis

Probabilistic sensitivity analyses (PSA) were performed using 1 000 simulations to quantify the parameter uncertainty around outcomes of the base case and scenarios 1–4. In each simulation, complication probabilities and costs were varied simultaneously with 20% variability, sampled from beta and gamma distributions respectively. This level of variability was applied in line with guidelines for conducting economics analyses for parameters for which uncertainty is not well established [37]. In scenario analyses 1–4, TEVG-specific complication probabilities and unit costs were fixed, reflecting scenario-specific assumptions, while ePTFE graft probabilities and shared costs were varied. Results are presented as 95% uncertainty intervals (UI) for base case outcomes and scenario break-even prices. Importantly, assumptions underlying the parameter distributions also remain uncertain. Therefore, the UI should be interpreted as indicative of the potential range, rather than as precise estimates of decision uncertainty.

### 2.6. Ethics and data management

The study was reviewed through the institutional ethics procedure and did not fall under the Dutch Medical Research Involving Human Subjects Act (23U-0279). Therefore, it did not require approval from an ethics review committee.

## 3. Results

### 3.1. Patient journey

The patient journey of Dutch VA care consists of four care phases: 1) creating access, 2) pre-dialysis, 3) hemodialysis without complications, and 4) hemodialysis with complications and interventions (Figure 2). In each phase, care activities could be performed by one or multiple specialists: 1) nephrologists, 2) vascular surgeons, 3) intervention radiologists, and 4) dialysis nurses. Care phases are depicted vertically, while care activities performed by different specialties are depicted horizontally. Information saturation was reached after seven interviews with nephrologists (*N* = 2), dialysis nurses (*N* = 2), a vascular surgeon (*N* = 1), an intervention radiologist (*N* = 1), and a patient (*N* = 1). Stakeholders did not anticipate differences in the type of care utilization between ePTFE grafts and the different TEVGs. Thus, the VA care pathway for grafts is considered universal.

**Figure 2.**
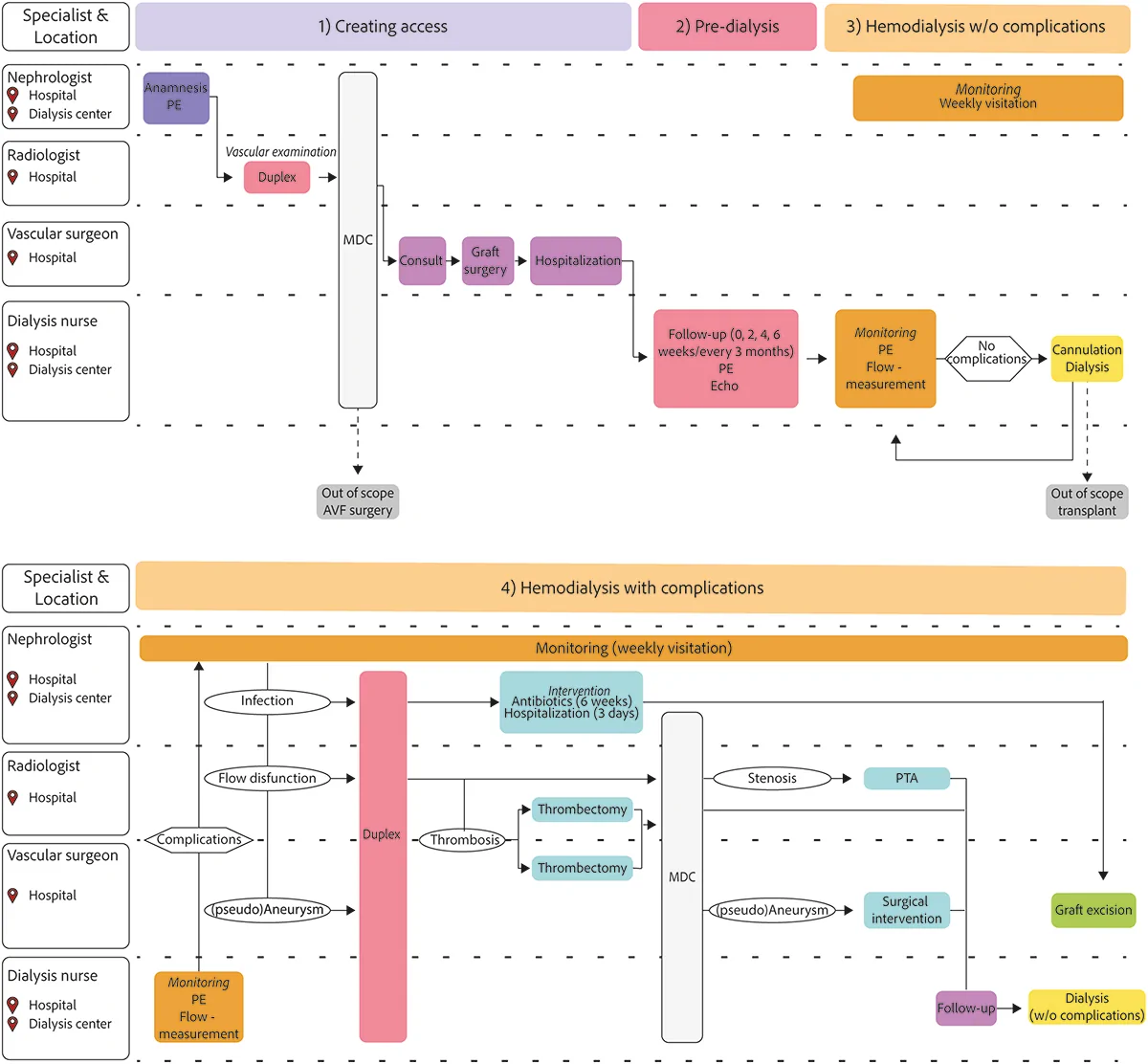
Patient journey displaying vascular access (VA) creation via arteriovenous (AV) grafts, graft monitoring and management.

The horizontal lanes represent medical specialties involved with the care activities, while columns represent the different care phases in the hemodialysis care pathway.

Abbreviations: PE = physical examination, MDC = multidisciplinary consultation, PTA = percutaneous transluminal angioplasty.

### 3.2. Early economic evaluation

#### 3.2.1. Base case

The cohort distribution across states over 12 months is depicted in Figure 3. After 12 months, 35% and 43% of the cohort reside in the functional state for ePTFE grafts and TEVGs, respectively. In these 12 months, ePTFE grafts required 2.46 interventions (95% UI 2.08–2.78), including infectious treatment, PTA procedures, radiological thrombectomy, stenting and surgical revision, at a total cost of €7 438 (95% UI €5 922 – €9 072) per patient. Point estimates are deterministic base case outcomes, while UI reflect PSA results. The combined TEVG base case (50% reduction in infection treatments and PTAs) required 2.02 interventions (95% UI 1.70–2.24), costing €6 837 (95% UI €5 426 – €8 483). These results reflect 0.44 fewer interventions (95% UI −0.91 – +0.03) and €601 savings (95% UI –€1 864 – +€647) per patient. Interventions were aggregated into one intervention state, so individual intervention frequency could not be separately identified. The distribution of PSA iterations is presented in Supplementary Figure S1.

**Figure 3.**
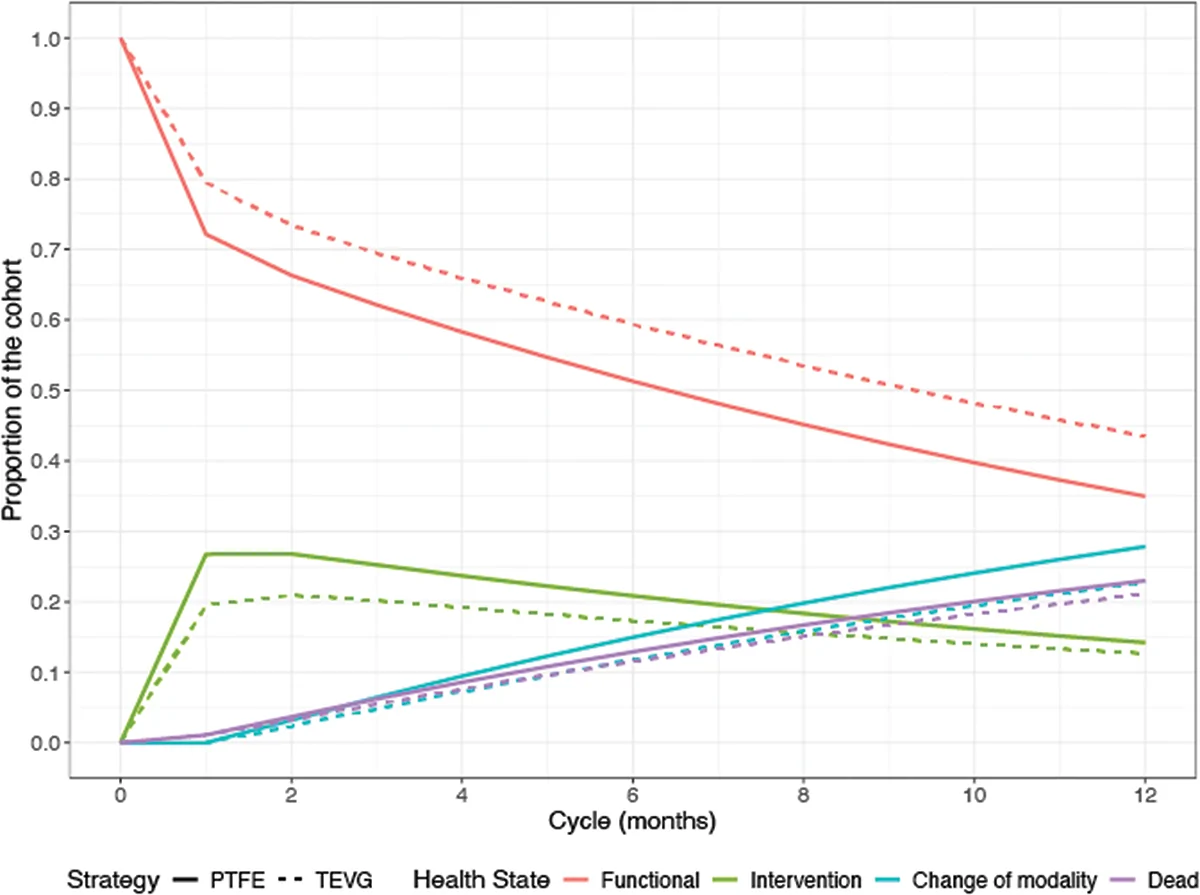
Proportion of the cohort residing in the different health states over time (12 months) for patients receiving expanded polytetrafluoroethylene (ePTFE) grafts and tissue engineered vascular access grafts (TEVGs).

Solid lines represent ePTFE grafts and dashed lines represent TEVGs. The functional health state is depicted in red, intervention health state in green, graft abandonment in blue and death in purple.

#### 3.2.2. Scenario and sensitivity analyses

As TEVGs’ intervention rates and unit prices remain uncertain, the impact of varying these factors on total intervention frequency and costs was explored across four clinical scenarios (Table 3). The outcomes of each scenario were compared to ePTFE grafts (Table 4). Scenarios that show higher overall costs are highlighted in red.

**Table 4.** Outcomes of scenario analyses for Tissue engineered Vascular access Grafts (TEVGs) compared to expanded polytetrafluoroethylene (ePTFE).

Scenario	TEVG price €1 000	TEVG price €1 250	TEVG price €1 500	TEVG price €3 000	TEVG price €3 500	Intervention rate	Absolute intervention reduction	Estimated Break-even point (TEVG price)
								€ 3 349
								(95% UI
1 (best-case)	€ 5 089	€ 5 339	€ 5 589	€ 7 089	***€ 7 589***	1.49	0.97	€1 675 – €5 349)
								€ 1 996
								(95% UI
2 (50% of best-case	€ 6 442	€ 6 692	€ 6 942	***€ 8 442***	***€ 8 942***	2.05	0.41	€530 – €3 492)
								€ 1 452
								(95% UI
3 (25% of best-case)	€ 6 986	€ 7 236	***€ 7 486***	***€ 8 986***	***€ 9 486***	2.27	0.19	-€4 – €2 777)
								€ 1 159
								(95% UI
4 (10% of best-case)	€ 7 279	***€ 7 529***	***€ 7 779***	***€ 9 279***	***€ 9 779***	2.39	0.07	-€291 – €2 496)

Scenarios that are more costly than the ePTFE base case (€7 438) are highlighted in bold and italic. Interventions include infectious treatment, percutaneous transluminal angioplasty (PTA) procedures, radiological thrombectomy, stenting and surgical revision.

Abbreviations: UI = uncertainty interval.

The deterministic best-case scenario (scenario 1) resulted in a reduction of 0.97 interventions per year under budget-neutral TEVG-introduction at a unit price up to €3 349 (95% UI €1 675 – €5 349). In scenario 2, the intervention rate decreased by 0.41 per year, while maintaining budget neutral up to a unit price of €1 996 (95% UI €530 – €3 492). Scenarios 3 and 4 showed 0.19 and 0.07 fewer interventions and remained budget neutral up to unit prices of €1 452 (95% UI –€4 – +€2 777) and €1 159 (95% UI –€291 – +€2 496), respectively (Table 4).

To further investigate the effect of individual parameters on the total costs of the VA graft and graft management pathway, a DSA was performed in which individual parameters were varied between 50% (min) and 150% (max) from the base case scenario. The resulting cost differences are visualized in a tornado plot, where parameters are ranked in descending order of impact on total costs (Figure 4). The sensitivity analysis indicates that alterations in the frequency of thrombosis, initial cost of TEVGs and a reduction of stenotic complications are the biggest drivers of costs in this study population.

**Figure 4.**
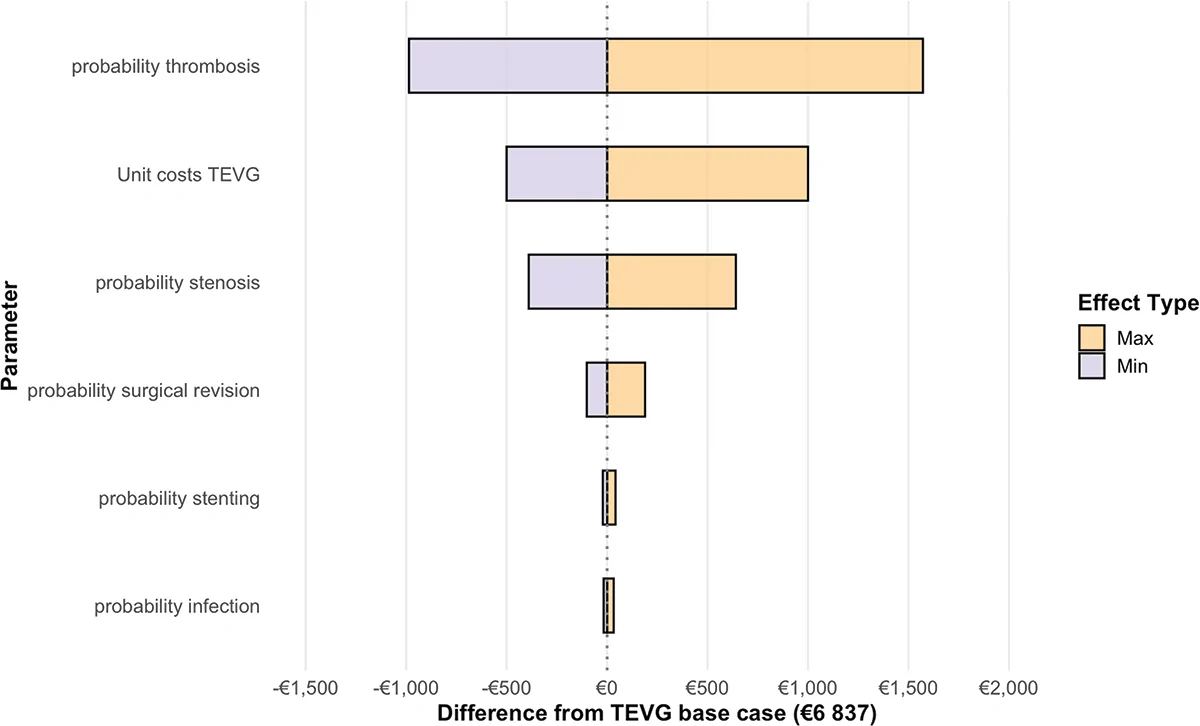
Tornado diagram of deterministic sensitivity analysis (DSA).

The range of cost differences in euros (x-axis) when the transition probabilities of interventions and the costs of Tissue Engineered Vascular Grafts (TEVG) are varied between 50% (min, purple) and 150% (max, yellow) of the TEVG base case scenario.

## 4. Discussion

This early economic evaluation quantified clinical and economic thresholds to inform clinical performance and design requirements for TEVGs, enabling them to become viable alternatives to ePTFE grafts in ESKD patients undergoing hemodialysis in the Netherlands. Model outcomes should not be interpreted as firm economic evidence of cost-effectiveness of TEVGs but represent model-based projections as a tool to prioritize research efforts and guide further graft development to increase translational success of TEVGs. To this end, several scenarios assessed the potential impact on intervention frequency and costs of TEVGs on the VA care pathway. In the combined TEVG base-case analysis, assuming a 50% reduction in infection and PTA procedures compared to ePTFE grafts, the annual intervention rate decreased from 2.46 to 2.02, yielding cost savings of €601. Scenario analyses, from best-case to less optimistic assumptions, produced intervention rates ranging from 1.49 to 2.39 per year, with break-even TEVG unit prices between €3 349 and €1 159. These findings indicate the clinical performance and cost thresholds TEVGs must meet to become a viable alternative to ePTFE grafts within current VA care budgets.

Comparison of our thresholds to early pre-clinical results from literature provides an initial indication of how TEVGs may perform relative to these thresholds. While studies in large animals with *in vivo* and *in situ* TEVGs show similar or worse clinical outcomes compared to ePTFE grafts, a phase II clinical trial with *in vitro* TEVGs reported yearly intervention rates of 1.67 and 1.95 for two different cohorts [13,17,19,20]. Now that clinical threshold ranges are defined, these early pre-clinical results offer valuable insight into the feasibility of specific TEVG innovations. Further evidence is expected for both *in situ* and *in vitro* grafts [38].

Beyond mean intervention rate, sensitivity analyses identified the incidence of thrombosis, stenosis and TEVG unit price as key parameters influencing economic feasibility. In literature, thrombosis and stenosis remain frequently reported complications [13,15,17–20]. Consequently, TEVG economic competitiveness will likely depend on demonstrating the incidence of thrombosis and stenosis, as well as production costs and subsequent pricing of TEVGs.

In this analysis, three types of TEVGs (*in vivo, in vitro* and *in situ*) were grouped, as clinical effectiveness and costs currently cannot be reliably distinguished. Consequently, clinical performance was assumed to be similar across TEVG types, yet as these technologies further mature, differences in clinical outcomes, care and costs can be expected. For example, cell‑based *in vitro* strategies entail lengthier and labor-intensive production procedures, while *in vivo* grafts may require additional implantation surgery. Since thresholds for competing with ePTFE grafts are set the same for all TEVGs, each type must balance achievable intervention reduction with an acceptable unit price. However, better clinical effectiveness may justify higher graft price and healthcare cost, particularly if improved outcomes translate into reduced long-term complications or healthcare utilization.

### 4.1. Strengths and limitations

To our knowledge, this study represents the first early economic evaluation of TEVGs in ESKD patients undergoing hemodialysis. As such, it illustrates how early economic modeling can be integrated parallel to product development to guide ongoing development of biomedical innovations. Our model reflects a VA graft care pathway that is considered universal across different graft types, enabling scenario exploration for different TEVGs and other innovations in the VA graft field and can be updated once clinical data emerges, allowing assessment of novel grafts. Model limitations must be considered when interpreting the results. This study aims to inform product development and strategic decision-making rather than to evaluate clinical effectiveness. As is typical for these evaluations, robust clinical data was not available. Therefore, transition probabilities were derived from a published model and supplemented with intervention rates from a cohort study [9,31]. Transition probabilities from the model considered only severe complications, whereas the cohort also included milder ones. Combining these sources may have led to underestimating recovery and overestimating modality changes, potentially resulting in patients leaving the model prematurely. As these patients are no longer at risk of contracting complications, this might have resulted in an underestimation of intervention rates and associated costs. This occurrence should be more profound in scenarios with higher complication rates, suggesting that lower intervention rates and costs are estimated for ePTFE grafts than for TEVG scenarios. To validate our results, clinicians reviewed the cohort’s progression through our model and confirmed that these patterns were clinically plausible. However, availability of real-world data will enable calculation of transition probabilities which will provide a more accurate reflection. Furthermore, these study outcomes reflect complication rates and costs from the Dutch healthcare system. While the Dutch VA care pathway largely aligns with European and American clinical guidelines, intervention frequencies, associated costs, and clinical outcomes may vary across health systems [3,27,28]. For example, meta-analyses report higher infection rates in ePTFE grafts than those observed in the Netherlands [9,39]. Potentially, higher baseline complication rates suggest that greater health benefits and cost-savings may be achievable in those settings. Given that the care pathway is generalizable across settings, the model structure can be easily adapted to different countries, allowing assessment of TEVGs in diverse (non-Dutch) healthcare settings.

### 4.2. Implications and future directions

The thresholds identified in this analysis can serve as practical reference points for developers to guide and prioritize further research efforts and to support early, informed discussions with payers around value and willingness to pay. Key research areas include reducing the incidence of thrombosis and stenosis, as well as TEVG manufacturing costs and pricing strategies. As clinical trials progress beyond the proof-of-concept stage, the findings of this study highlight that future trials should, at a minimum, capture complication rates alongside cost-consequences of TEVG use. In addition, data on long-term healthcare utilization, graft durability, patient survival, quality of life, and patient preferences should be collected to accurately shape and assess the value proposition of TEVGs. Mature data including these measures can be used in future economic evaluation to assess TEVGs clinical and economic impact. These evaluations should also continue to incorporate robust sensitivity analyses to characterize remaining uncertainty.

## 5. Conclusion

This early economic evaluation estimated the clinical and economic thresholds of TEVGs to compete with ePTFE grafts based on current evidence. The analysis provides model-based projections to prioritize research directions in further TEVG development. TEVGs have the potential to become clinically and economically competitive alternatives to ePTFE grafts for ESKD patients requiring hemodialysis. Their competitiveness will depend on sufficiently reducing intervention rates, especially thrombectomies and PTA procedures, and production costs and pricing. Future research and development efforts should prioritize strategies to minimize stenotic and thrombotic complications during clinical development in addition to rigorous evaluation of manufacturing costs and pricing strategies to ensure economic viability.

## Supplementary Material

Supplementary_figure_1.tif

Supplementary_table doc.docx

## Data Availability

The data underlying this article are available in the article.

## Article highlights

- Tissue-engineered vascular access grafts (TEVGs) are emerging as a promising alternative to current vascular access options for hemodialysis patients with end-stage kidney disease.
- The clinical performance and cost thresholds required for TEVGs to become a competitive and economically viable alternative to expanded polytetrafluoroethylene (ePTFE) grafts, which are the most closely related current standard, in routine hemodialysis care have not previously been quantified.
- This early economic evaluation quantifies these thresholds, calculates potential break-even TEVG unit prices, and identifies key cost drivers through patient-journey mapping and Markov modeling.
- In the absence of mature clinical data, initial estimates on TEVG performance, best-case and progressively less optimistic scenarios quantified the potential health benefits and cost-consequences of TEVGs.
- TEVGs have the potential to be clinically and economically competitive with ePTFE grafts. However, their competitiveness will depend critically on reducing thrombectomy and percutaneous transluminal angioplasty (PTA) rates and achieving cost-efficient TEVG unit pricing.
- Further research should therefore prioritize collecting data on thrombosis and stenosis as well as gaining detailed insight into TEVG production costs and pricing strategies.
- These findings provide actionable performance benchmarks to guide further TEVG development, while informing developers, clinicians and decision-makers on their value proposition.
- This early-economic model is designed to evolve alongside TEVG development, as it can be easily updated as clinical data matures and more evidence becomes available.

## References

[1] Foreman KJ, Marquez N, Dolgert A, et al. Forecasting life expectancy, years of life lost, and all-cause and cause-specific mortality for 250 causes of death: reference and alternative scenarios for 2016–40 for 195 countries and territories. Lancet. 2018;392(10159):2052–2090. doi: 10.1016/S0140-6736(18)31694-5 PMC6227505 30340847

[2] Bello AK, Okpechi IG, Levin A, et al. ISN–global kidney health atlas: a report by the International society of nephrology: an assessment of global kidney health care status focussing on capacity, availability, accessibility, affordability and outcomes of kidney disease. 2023. https://www.theisn.org/in-action/research/global-kidney-health-atlas/

[3] Lok CE, Huber TS, Lee T, et al. KDOQI clinical practice guideline for vascular access: 2019 update. Am J Kidney Dis. 2020;75(4 Suppl 2):S1–s164. 10.1053/j.ajkd.2019.12.001 32778223

[4] Almasri J, Alsawas M, Mainou M, et al. Outcomes of vascular access for hemodialysis: a systematic review and meta-analysis. J Vasc Surg. 2016;64(1):236–243. doi: 10.1016/j.jvs.2016.01.053 27345510

[5] Lee T. Fistula first initiative: historical impact on vascular access practice patterns and influence on future vascular access care. Cardiovasc Eng Technol. 2017;8(3):244–254. doi: 10.1007/s13239-017-0319-9 PMC5693683 28695442

[6] Voorzaat BM, van der Bogt KEA, Janmaat CJ, et al. Arteriovenous fistula maturation failure in a large cohort of hemodialysis patients in the Netherlands. World J Surg. 2018;42(6):1895–1903. doi: 10.1007/s00268-017-4382-z PMC5934452 29188313

[7] Huber TS, Berceli SA, Scali ST, et al. Arteriovenous fistula maturation, functional patency, and intervention rates. JAMA Surg. 2021;156(12):1111–1118. doi: 10.1001/jamasurg.2021.4527 PMC8459303 34550312

[8] Gallieni M, Brenna I, Brunini F, et al. Dialysis central venous catheter types and performance. J Vasc Access. 2014;15 Suppl 7(7_suppl):S140–6. doi: 10.5301/jva.5000262 24817472

[9] Voorzaat BM, Janmaat CJ, van der Bogt KEA, et al. Patency outcomes of arteriovenous fistulas and grafts for hemodialysis access: a trade-off between nonmaturation and long-term complications. Kidney360. 2020;1(9):916–924. doi: 10.34067/KID.0000462020 PMC8815607 35369548

[10] Benrashid E, McCoy CC, Youngwirth LM, et al. Tissue engineered vascular grafts: origins, development, and current strategies for clinical application. Methods. 2016;99:13–19. doi: 10.1016/j.ymeth.2015.07.014 26216054

[11] Lawson JH, Niklason LE, Roy-Chaudhury P. Challenges and novel therapies for vascular access in haemodialysis. Nat Rev Nephrol. 2020;16(10):586–602. doi: 10.1038/s41581-020-0333-2 PMC8108319 32839580

[12] Dahl SL, Kypson AP, Lawson JH, et al. Readily available tissue-engineered vascular grafts. Sci Transl Med. 2011;3(68):68ra9. 10.1126/scitranslmed.3001426 21289273

[13] Geelhoed WJ, van der Bogt KEA, Rothuizen TC, et al. A novel method for engineering autologous non-thrombogenic in situ tissue-engineered blood vessels for arteriovenous grafting. Biomaterials. 2020;229:119577. doi: 10.1016/j.biomaterials.2019.119577 31704466

[14] Nakayama Y, Furukoshi M, Terazawa T, et al. Development of long in vivo tissue-engineered “biotube” vascular grafts. Biomaterials. 2018;185:232–239. doi: 10.1016/j.biomaterials.2018.09.032 30248647

[15] Ebner A, Gallo S, Castellano J, et al. First-in-human evaluation of a biological regenerative vascular conduit for hemodialysis access. J Vasc Access. 2023;25(4):1271–1278. doi: 10.1177/11297298221147709 36895135

[16] Blum KM, Zbinden JC, Ramachandra AB, et al. Tissue engineered vascular grafts transform into autologous neovessels capable of native function and growth. Commun Med (Lond). 2022;2(1):3. doi: 10.1038/s43856-021-00063-7 PMC9053249 35603301

[17] Syedain ZH, Graham ML, Dunn TB, et al. A completely biological “off-the-shelf” arteriovenous graft that recellularizes in baboons. Sci Transl Med. 2017;9(414). doi: 10.1126/scitranslmed.aan4209 29093182

[18] Jakimowicz T, Przywara S, Turek J, et al. Five year outcomes in patients with end stage renal disease who received a bioengineered human acellular vessel for dialysis access. EJVES Vasc Forum. 2022;54:58–63. doi: 10.1016/j.ejvsvf.2022.01.003 PMC8881722 35243473

[19] Lawson JH, Glickman MH, Ilzecki M, et al. Bioengineered human acellular vessels for dialysis access in patients with end-stage renal disease: two phase 2 single-arm trials. Lancet. 2016;387(10032):2026–2034. doi: 10.1016/S0140-6736(16)00557-2 PMC4915925 27203778

[20] Besseling PJ, Szymczyk W, Teraa M, et al. Off-the-shelf synthetic biodegradable grafts transform in situ into a living arteriovenous fistula in a large animal model. Adv Healthc Mater. 2024;13(17):e2303888. doi: 10.1002/adhm.202303888 PMC11469054 38451476

[21] Gardner J, Faulkner A, Mahalatchimy A, et al. Are there specific translational challenges in regenerative medicine? Lessons from other fields. Regen Med. 2015;10(7):885–895. doi: 10.2217/rme.15.50 26541074

[22] Gardner J, Webster A. The social management of biomedical novelty: facilitating translation in regenerative medicine. Soc Sci Med. 2016;156:90–97. doi: 10.1016/j.socscimed.2016.03.025 27019143

[23] West-Livingston L, Lim JW, Lee SJ. Translational tissue-engineered vascular grafts: from bench to bedside. Biomaterials. 2023;302:122322. doi: 10.1016/j.biomaterials.2023.122322 37713761

[24] Ijzerman MJ, Steuten LM. Early assessment of medical technologies to inform product development and market access: a review of methods and applications. Appl Health Econ Health Policy. 2011;9(5):331–347. 10.2165/11593380-000000000-00000 21875163

[25] Ijzerman MJ, Koffijberg H, Fenwick E, et al. Emerging use of early health technology assessment in medical product development: a scoping review of the literature. Pharmacoeconomics. 2017;35(7):727–740. doi: 10.1007/s40273-017-0509-1 PMC5488152 28432642

[26] Bulto LN, Davies E, Kelly J, et al. Patient journey mapping: emerging methods for understanding and improving patient experiences of health systems and services. Eur J Cardiovasc Nurs. 2024;23(4):429–433. doi: 10.1093/eurjcn/zvae012 38306596

[27] Federatie van Medische Specialisten. Vaattoegang voor hemodialyse 2022 [updated 2022 Feb 24; cited 2023]. Available from: https://richtlijnendatabase.nl/richtlijn/vaattoegang_voor_hemodialyse/operatieve_besluitvorming_bij_vaattoegang_voor_hemodialyse/soort_vaattoegang_en_locatie_voor_hemodialyse.html

[28] Schmidli J, Widmer MK, Basile C, et al. Editor’s choice - vascular access: 2018 clinical practice guidelines of the European society for vascular surgery (ESVS). Eur J Vasc Endovasc Surg. 2018;55(6):757–818. 10.1016/j.ejvs.2018.02.001 29730128

[29] Laari L. Qualitative data saturation in health sciences research. Nurse Res. 2025;33(4):32–39. doi: 10.7748/nr.2025.e1948 41164886

[30] Alarid-Escudero F, Krijkamp E, Enns EA, et al. An introductory tutorial on cohort state-transition models in R using a cost-effectiveness analysis example. Med Decis Mak. 2023;43(1):3–20. doi: 10.1177/0272989X221103163 PMC9742144 35770931

[31] Leermakers JJ, Bode AS, Vaidya A, et al. Cost-effectiveness of vascular access for haemodialysis: arteriovenous fistulas versus arteriovenous grafts. Eur J Vasc Endovasc Surg. 2013;45(1):84–92. doi: 10.1016/j.ejvs.2012.10.012 23153926

[32] Nefrovisie. Renine registry annual report 2021. 2021.

[33] Nederlandse Zorgautoriteit. [cited 2024 Aug 26]. Available from: https://www.opendisdata.nl

[34] Hakkaart-van Roijen LV, Bouwmans CAM, Kanters TA, et al. Costing manual: methodology of costing research and reference prices for economic evaluations in healthcare. Zorginstituut Nederland; 2016. Available from: https://www.zorginstituutnederland.nl/publicaties/publicatie/2016/02/29/richtlijn-voor-het-uitvoeren-van-economische-evaluaties-in-de-gezondheidszorg

[35] Nederlandse Zorgautoriteit. Prijsindexcijfers 2024. [cited 2024 Aug 26]. Available from: https://www.nza.nl/onderwerpen/prijsindexcijfers

[36] Wijnen E, Planken N, Keuter X, et al. Impact of a quality improvement programme based on vascular access flow monitoring on costs, access occlusion and access failure. Nephrol Dial Transpl. 2006;21(12):3514–3519. doi: 10.1093/ndt/gfl424 16921189

[37] Nederlandse Zorgauthoriteit. Richtlijn voor het uitvoeren van economische evaluaties in de gezondheidszorg: herziene versie 2024. Diemen: Zorginstituut Nederland; 2024.

[38] De Vriese AS, D’Haeninck A, Mendes A, et al. Study protocol of a prospective single-arm multicenter clinical study to assess the safety and performance of the aXess hemodialysis graft: the pivotal study. J Vasc Access. 2024;25(4):1301–1307. doi: 10.1177/11297298231174932 37165650

[39] Halbert RJ, Nicholson G, Nordyke RJ, et al. Patency of ePTFE arteriovenous graft placements in hemodialysis patients: systematic literature review and meta-analysis. Kidney360. 2020;1(12):1437–1446. doi: 10.34067/KID.0003502020 PMC8815525 35372887

