# Supplementary material for: Early economic evaluation of tissue engineered vascular access grafts for hemodialysis
Source: Regen Med. 2026 Sep 21;21(8-9):469–78. doi: 10.1080/17460751.2026.2726833 (PMC13671427; doi:10.1080/17460751.2026.2726833)
Supplement: Supplementary file 1 [file IRME_A_2726833_SM3372.docx]

| **Complication rates AVF** | | | | |
| --- | --- | --- | --- | --- |
| **Complication type** | **Complication rate AVF Radiocephalic** | **Complication rate AVF  Upper arm** | **Complication rate  average** | **Source** |
| Infection | 0.005 | 0.070 | 0.037 | [9] |
| PTA | 1.010 | 1.640 | 1.325 | [9] |
| Thrombectomy | 0.030 | 0.050 | 0.040 | [9] |
| Surgical revision | 0.290 | 0.050 | 0.170 | [9] |

**Supplementary table 1A**

**Supplementary table 1B**

| **Infection** | | | | | |
| --- | --- | --- | --- | --- | --- |
| **Infection** | **Difference in rate** | | **New rate** | **New transition probability** | **Percentage reduction** |
| 1 (best-case = AVF rate) | | 0.063 | 0.037 | 0.003 | 63% |
| 2 (50% of best-case) | | 0.031 | 0.069 | 0.006 | 31% |
| 3 (25% of best-case) | | 0.016 | 0.084 | 0.007 | 16% |
| 4 (10% of best-case) | | 0.006 | 0.094 | 0.008 | 6% |
| **PTA** | | | | | |
| **Reduction** | **Difference in rate** | | **New rate** | **New transition probability** | **Percentage reduction** |
| 1 (best-case = AVF rate) | | 0.515 | 1.325 | 0.105 | 28% |
| 2 (50% of best-case) | | 0.258 | 1.583 | 0.124 | 14% |
| 3 (25% of best-case) | | 0.129 | 1.711 | 0.133 | 7% |
| 4 (10% of best-case) | | 0.052 | 1.789 | 0.138 | 3% |
| **Thrombectomy** | | | | | |
| **Reduction** | **Difference in rate** | | **New rate** | **New transition probability** | **Percentage reduction** |
| 1 (best-case = AVF rate) | | 1.090 | 0.040 | 0.003 | 96% |
| 2 (50% of best-case) | | 0.545 | 0.585 | 0.048 | 48% |
| 3 (25% of best-case) | | 0.273 | 0.858 | 0.069 | 24% |
| 4 (10% of best-case) | | 0.109 | 1.021 | 0.082 | 10% |
| **Surgical revision** | | | | | |
| **Reduction** | **Difference in rate** | | **New rate** | **New transition probability** | **Percentage reduction** |
| 1 (best-case = AVF rate) | | 0.140 | 0.170 | 0.014 | 45% |
| 2 (50% of best-case) | | 0.070 | 0.240 | 0.020 | 23% |
| 3 (25% of best-case) | | 0.035 | 0.275 | 0.023 | 11% |
| 4 (10% of best-case) | | 0.014 | 0.296 | 0.024 | 5% |
